# Supplemental organic trace minerals and a yeast culture product in newly weaned steers: effects of use and delivery method on growth performance and hepatic trace mineral content^1^

**DOI:** 10.1093/tas/txad119

**Published:** 2023-10-18

**Authors:** Thiago Lauro Maia Ribeiro, Forest L Francis, Erin R Gubbels, Jason E Griffin, Warren C Rusche, Zachary K Smith

**Affiliations:** Department of Animal Science, South Dakota State University, Brookings, SD 57007; Department of Animal Science, South Dakota State University, Brookings, SD 57007; Department of Animal Science, South Dakota State University, Brookings, SD 57007; Department of Animal Science, South Dakota State University, Brookings, SD 57007; Department of Animal Science, South Dakota State University, Brookings, SD 57007; Department of Animal Science, South Dakota State University, Brookings, SD 57007

**Keywords:** feedlot, receiving period, trace mineral, yeast culture product

## Abstract

The objective of this study was to determine if supplementation and delivery method of a “stress pack” composed of organic trace minerals and *Saccharomyces cerevisiae* yeast culture product influenced growth performance, feed efficiency, and hepatic trace mineral concentration in newly weaned steers. Crossbred steers (*n* = 192; 256 ± 14.0 kg) were used in a 49-day receiving phase experiment. Within 36 hours of weaning, steers were weighed, allotted to 24 pens (*n* = 8 steers/pen; 8 pens/treatment), and randomly assigned to treatments: 1) a traditional receiving diet (**CON**), 2) a traditional receiving diet plus the “stress-pack” directly in the diet (FORCE), and 3) a traditional receiving diet plus a low-moisture, cooked molasses block fortified with the “stress-pack” (**TUB**). The “stress-pack” was offered the first 28 day of the 49-day receiving period. Due to adverse weather conditions forecasted on day 1, biopsy samples were collected from a subsample of steers (*n* = 14 steers) on day 1 to establish hepatic trace mineral concentration baseline. Steers were selected based on the mean body weight (BW) from allotment (day −1) of the pen for collection of subsequent samples (*n* = 1 steer/pen) on days 14, 28, and 49 for hepatic trace mineral concentration determination. Cumulative dry matter intake (DMI) (*P* = 0.01) was greater for FORCE compared to CON and TUB. Final BW and average daily gain (ADG) tended (*P* ≤ 0.10) to be greater for FORCE compared to TUB and CON by 5.4% and 9.4%, respectively. Feed efficiency did not differ between treatments (*P* = 0.28). A treatment × day interaction (*P* ≤ 0.01) for hepatic Cu concentration was noted. The FORCE treatment had greater hepatic Cu compared to TUB and CON for the entire period. The steers that received TUB had greater hepatic Cu compared to CON on days 14 and 28, but similar to CON on day 49. The addition of a “stress-pack” to diets offered to newly weaned cattle enhanced hepatic trace mineral concentration, and delivery method influences DMI and daily gain.

## Teaser

The present study was conducted to evaluate the effects of delivery method of oragnic trace minerals and yeast culture product(“stress-pack”) has on production efficiency and hepatic trace mineral status and demonstrated that method of delivery alters intake and hepatic trace mineral status.

## Introduction

Weaning and transportation are known to be highly stressful events in the life of a beef calf. Road transportation involves loading the animals at their place of origin, confining them on a moving vehicle, depriving them of feed and water, and then unloading and penning them at their destination. Upon arrival, they encounter different sources of feed and water and are often comingled with other animals. As a result, these experiences lead to various changes and challenges in their immune, hormonal, physiological, and nutritional status (Loerch and Fluharty, 1999; Earley et al., 2012). Moreover, stress has a significant impact on the trace mineral (**TM**) status of cattle (NASEM, 1996). This is because stress triggers the mobilization of tissue reserves of essential elements such as Co, Cu, Mn, and Zn, which are crucial for supporting immune function. This effect is particularly pronounced in newly received animals (Duff and Galyean, 2007).

*Saccharomyces cerevisiae* yeast culture products have long been used in domestic animal diets and have been shown to positively influence the ruminal environment and stimulate the growth of beneficial microorganisms (Callaway and Martin, 1997; Miller-Webster et al., 2002; Geng et al., 2016). *Saccharomyces cerevisiae* yeast culture products are composed of byproducts of the fermentation process, including B-vitamins, amino acids, and nucleic acids (Callaway and Martin, 1997; Wagner et al., 2016; Mohammed et al., 2018). Studies (Geng et al., 2016; Alugongo et al., 2017; Mohammed et al., 2018) suggest that yeast culture products (**YCP**) have positive effects on growth performance, rumen, intestinal health, and immune responses. In addition, cattle-fed YCP also exhibited improved fiber digestibility, mineral retention, and flow of microbial protein to the small intestine (Cole et al., 1992; Newbold et al., 1996; Mohammed et al., 2018).

Supplementation of TM and YCP in combination has led to greater performance and greater hepatic TM content compared to nontreated steers (Hamilton et al., 2021). However, different delivery methods of these products when combined have not been evaluated in a feedlot setting. Therefore, the objective of this study was to determine if use and method of delivery of added organic TM (Availa 4, ZINPRO, Eden Prairie, MN) and a *Saccharomyces cerevisiae* YCP (Diamond V XPC, Diamond V, Cedar Rapids, IA) fed in combination (stress-pack) affect growth performance and hepatic trace mineral concentration upon feedlot introduction in newly weaned beef steer calves. We hypothesized that additional TM and YCP would increase daily intake and gain, and method of supplementation would have no influence on production outcomes.

## Methods and Materials

### Use of Animal Subjects

All procedures involving the use of animals in this experiment were approved by the South Dakota State University Institutional Animal Care and Use Committee (Approval #2108-049A).

### Animal Description and Initial Processing

Charolais × Angus steers calves (*n* = 192; 256 ± 14.0 kg) from a single source were transported (515 km) to the Ruminant Nutrition Center in Brookings, SD. Upon arrival (6.5 hours transport from the ranch), steers were offered long-stem grass hay and ad libitum access to water. The following day (day −1), steers were weighed, tagged, and vaccinated (Bovi-Shield GOLD 5, Zoetis, Parsippany, NJ; Ultrabac 7/Somubac, Zoetis), administered a dose of pour-on moxidectin (Cydectin, Bayer Animal Health, Shawnee Mission, KS), and sorted into study pens the afternoon following initial processing (*n* = 8 steers per pen; 8 pens/treatment). Steers were individually weighed again on day 1. Initial BW was the average of the two BW measures collected on day −1 and day 1. Live BW measures, excluding initial BW, were shrunk 4% to account for digestive tract fill. Initial BW was not shrunk to accommodate for transportation shrink on day −1.

### Experimental Design and Treatments

This study used eight replicate pens per treatment, and each pen contained eight steers (*n* = 64 steers/treatment). Each pen was assigned to one of three dietary treatments in a randomized complete block design (**RCBD**), where location in the feedlot was considered the blocking factor. Dietary treatments included: 1) a traditional receiving diet (CON), 2) a traditional receiving diet plus the “stress-pack” directly in the diet (FORCE; Table 1), and 3) a traditional receiving diet plus a low-moisture, cooked molasses block fortified with the “stress-pack” (TUB; Table 2).

**Table 1.** The FORCE fed supplement formulation (as-is basis)^1^

Item	%, as-fed
Soybean hulls	63.94
Dry distillers grains plus solubles	16.90
*Saccharomyces cerevisiae* fermentation product ^2^	6.25
Beet molasses	5.00
Organic trace mineral product ^3^	3.10
Salt	1.75
Potassium chloride	1.29
Selenium.16%	0.81
Vitamin E 50	0.35
Zinc sulfate, ppm	2500.00
Manganese sulfate, ppm	1400.00
Copper sulfate, ppm	900.00
Vitamin A 650	0.07
EDDI 9.2%	0.05
Vitamin D3 500	0.01

^1^FORCE-fed supplement was included in the diet at 0.23g/head/day (as-fed basis);.

^2^Diamond V Original XPC; Cedar Rapids, IA.

^3^Availa 4; Zinpro, Eden Prairie, MN.

**Table 2.** Minimum guaranteed analysis (as-is basis) for the low-moisture, molasses-based block (Stress TUB; Purina Animal Nutrition, St. Louis, MO, USA)^1^

Item	%, as-fed
Crude protein, %	12.00
Crude fat, %	4.00
Crude fiber, %	4.00
Calcium, %	2.00
Phosphorus, %	1.00
Salt, %	1.50
Magnesium, %	1.00
Potassium, %	2.00
Manganese, ppm	1300.00
Cobalt, ppm	60.00
Copper, ppm	785.00
Iodine, ppm	40.00
Selenium, ppm	13.00
Zinc, ppm	2500.00
Vitamin A, IU/kg	440,920.00
Vitamin D3, IU/kg	49,603.50
Vitamin E, IU/kg	1763.68

^1^Each 0.227 kg (as-is) an organic trace mineral product that provided: 200.2 mg of manganese, 12.6 mg of cobalt, 126.0 mg of copper, and 360.5 mg of zinc in each in each 0.227 kg (Availa 4; Zinpro, Eden Prairie, MN) and 14 g of Saccharomyces cerevisiae fermentation product (Diamond V Original XPC; Cedar Rapids, IA).

### Dietary Management

The traditional receiving diet (Table 3) consisted of 40% wheat silage, 10% oat hay, 36% soybean hulls, 9% DDGS, and 5% liquid supplement on DM basis (15% CP; 59% NDF; NEm; NEg; 1.73 and 1.04 Mcal/kg, respectively). The liquid supplement provided to the diet (DM basis) 27.5 mg/kg monensin sodium and inorganic trace minerals: Co (0.20 mg/kg), Cu (10 mg/kg), Mn (20 mg/kg), and Zn (35 mg/kg) to meet the nutrient requirements for growing beef steers (NASEM, 2016).

**Table 3.** Actual diet formulation based upon weekly DM determinations of ingredients and nutrient composition based upon 49-d period composites of weekly ingredients and reconstructed diet composition (DM basis) for nutrient content determination^1^

Item	%, dry matter
Wheatlage, %	39.62
DDGS^2^, %	9.39
Oat Hay, %	10.18
Pelleted Soybean Hulls, %	35.69
Liquid Supplement ^3^, %	5.12
DM, %	51.37
CP ^4^, %	12.92
NDF ^5^, %	56.49
ADF ^6^, %	38.64
Ash, %	6.99
EE ^7^, %	2.62
NEm ^8^, Mcal/kg	1.73
NEg ^9^, Mcal/kg	1.04

^1^All values except for dry matter (DM) on a DM basis.

^2^Dried distillers grains plus solubles.

^3^Contained (DM basis) 36.27% crude protein, 28.00% nonprotein nitrogen, 0.74 Mcal/kg of net energy for maintenance, 0.50 Mcal/kg of net energy for gain, 1.62% crude fat, 1.06% crude fiber, 4.62% calcium, 0.43% P, 2.28% K, 0.47% Mg, 5.00% NaCl, 3.38% Na, 0.54% S, 4.00 ppm Co, 200.00 ppm Cu, 20.00 ppm I, 25.15mg/kg. of ethylenediamine dihydroiodide 150.29 ppm Fe, 400.00 ppm Mn, 3.08 ppm Se, 700.00 ppm Zn, 44,092 IU/kg of vitamin A, 440.92 IU/kg of vitamin E, and 551.00 g/Mg of monensin sodium (Rumensin, Elanco, Indianapolis, IN, USA).

^4^Crude protein.

^5^Neutral detergent fiber.

^6^Acid detergent fiber.

^7^Ether extract.

^8^Net energy for maintenance.

^9^Net energy for gain.

All steers were fed twice daily (0800 hours and 1400 hours) in equal proportions. On day 1, 2 kg/steer of the diet (DM basis) was offered to the steers and increased by 0.5 kg/steer (DM basis) until day 7. Bunks were managed using a slick bunk approach from days 8 to 49 such that bunks were managed to be devoid of feed by 0730 hours. Pens were 7.6 × 7.6 m concrete surface pens with 7.6 m of linear bunk space and equipped with a heated, continuous flow concrete waterer.

Treatments were introduced approximately 36 hours following arrival to the RNC. Tubs (*n* = 1 tub/pen) were placed in the opposite corner of the feed bunk and waterer, tubs were replaced when 90% of the tub was consumed. Tubs were cleaned and weighed daily, and consumption was determined from daily disappearance. Individual consumption was calculated by dividing daily tub disappearance by the number of steers in the pen. Steers were provided access to the tubs through day 28 of the experiment. Force-fed supplement and Stress Tub nutritional values are presented in Tables 1 and 2, respectively. In addition, the FORCE supplement was mixed in the diet at a rate of 0.22 kg/steer·d^−1^. To facilitate the mixing process, a soybean hull carrier was used to ensure uniform distribution throughout the diet. The FORCE supplement was designed so that TM and YCP intake would be equivalent to tub assuming a targeted tub intake of 0.22 kg/steer·d^−1^. The FORCE supplements were included in the diet from day 1 to day 28.

Samples were collected weekly and dried in a forced air oven at 60°C until no further weight change occurred to determine DM content. Ingredients collected each week were ground to 1 mm and composited into a single individual sample for nutrient analyses at a commercial laboratory using (Servi-Tech, Hastings, NE) using AOAC procedures. Actual diet formulation based upon weekly DM determination and feed batching records along with tabular energy values (Preston, 2016) are presented in Table 3.

### Growth Performance Calculations

Steers were weighed before feeding, and on day −1, 1, 14, 28, and 49. Growth performance data were summarized from initial to d 49. Initial BW was not shrunk, while BW from day 49 was shrunk 4% to account for digestive tract fill. Average daily gain was determined using the difference in BW divided by days on feed. Efficiency of weight gain (G:F) was calculated by dividing the ADG by DMI.

### Hepatic Biopsies

For determination of hepatic trace mineral concentration, liver biopsies were collected on day −1, 14, 28, and 49 using the process described by Engle and Spears (2000). Due to adverse weather conditions forecasted on day 1, biopsy samples were collected from a subsample of steers (*n* = 14 steers) on day −1 to establish hepatic trace mineral concentration baseline. Steers were selected based on the mean BW from allotment of the pen for collection of subsequent samples (*n* = 1 steer/pen) on days 14, 28, and 49 for hepatic trace mineral concentration determination. Briefly, steers were secured in a hydraulic squeeze chute. The biopsy was collected through an incision in the 11th intercostal space on a line from the tuber coxae to the point of the scapula-humoral joint. Hair was clipped from an area approximately 10.16 cm × 10.16 cm around the biopsy site. The surgical site was prepared with iodine and 70% isopropyl alcohol. A solution composed of lidocaine hydrochloride and bicarbonate (90%/10%, v/v) was applied in the muscle layer and injected (1 mL) as the needle was withdrawn from the biopsy location The surgical site was prepared with iodine and 70% isopropyl alcohol. A solution composed of lidocaine hydrochloride and bicarbonate (90%/10%, v/v) was applied in the muscle layer and injected (1 mL) as the needle was withdrawn from the biopsy location. After 15 seconds, a 6.35 mm incision was made by inserting a scalpel blade through the skin and intercostal muscle. Surgical tubing was applied to the biopsy needle (DJ-series Jamshidi bone marrow needle 8 ga and 10.16 cm long; Cardinal Health catalog number DJ4008X 13), which was inserted into the incision site to reach the liver. The hepatic tissue was collected by using back pressure on a 10 mL syringe. Contents were emptied from surgical tub into a wire mesh screen, and blood was rinsed-off using 0.01M of phosphate buffered saline (Hamilton et al., 2021). Following this procedure there is no need for wound sealing due to the manner in which the insertions were made; gravity assists in naturally closing the wound. No complications were noted due to the hepatic biopsy procedure in the present study. Hepatic samples were shipped to Michigan State University Diagnostic Center for Population and Animal Health (Lansing, MI, USA) for analysis of hepatic mineral content. Concentrations of Co, Cu, Mn, and Zn were measured using an Agilent 7500ce Inductively Coupled Plasma Mass Spectrometer (Agilent Technologies Inc., Santa Clara, CA, USA) via procedures as described by (Wahlen et al., 2005).

### Statistical Analysis

Data were analyzed as a RCBD with pen as an experimental unit. Growth performance data were analyzed using the GLIMMIX procedure of SAS 9.4 (SAS Inst., Inc., Cary, NC, USA). Treatment was included as a fixed effect and block (location) was considered a random effect in the statistical model. Hepatic trace mineral content was analyzed via repeated measures and included the fixed effects of treatment, day, and their interaction, the covariance structure with the best fit (lowest Akaike information criterion) was autoregressive 1 (AR-1). Least squares means were generated and separated using pairwise comparisons. An *P* ≤ 0.05 determined significance, and an *P* = 0.06 to 0.10 was considered a tendency.

## Results and Discussion

**Figure 1.**
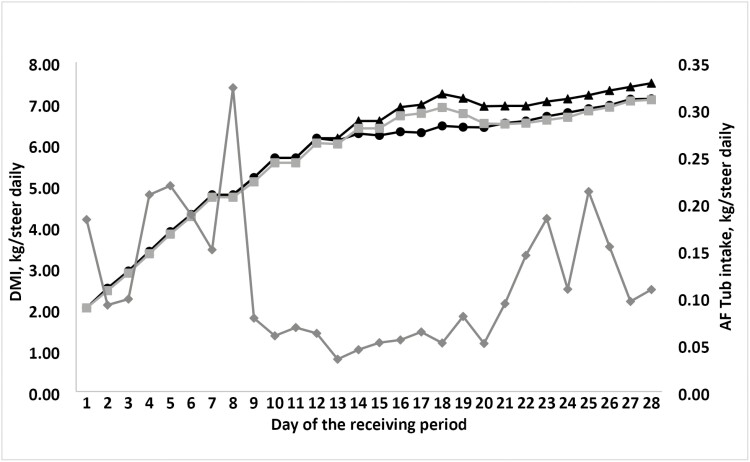
Dry matter intake and stress tub intake of newly weaned steers initial to 28-d post weaning. Daily (*n* = 8 pens) consumption (kg/steer·d^−1^) of the cooked molasses stress TUB (Stress TUB; Purina Animal Nutrition, St. Louis, MO, USA) during the 28-d period, offered to newly weaned steer calves. Dry matter intake (DMI; kg/steer daily), As-fed (AF) tub intake (kg/steer daily). Abbreviaitons: CON = black circle; FORCE = black triangle; TUB = gray square; AF tub consumption = gray diamond.

Tub disappearance and DMI are depicted in Figure 1. The stress tub label indicated that cattle should consume between 0.15 and 0.22 kg/d. Average daily disappearance from the tubs was 0.115 ± 0.06 kg/d, meaning that the steers under-consumed the supplement, a common issue associated with use of a free-choice supplement (Ranches et al., 2021). During the first 9 days, cattle consumed more of the tub, after which the consumption decreased (Figure 1). In addition, on day 9 steers reached DMI equivalent to 2% of BW, which is the desired DMI for receiving calves (Pritchard, 2005), and this likely contributed to the reduction in voluntary intake of the stress tub. A similar response was observed by Hamilton et al. (2021) where steers reduced the consumption of the stress tub on day 5.

Growth performance data are located in Table 4. Cumulative DMI of the total mixed ration (tub intake not included for TUB) was greater (*P* = 0.01) for FORCE compared to CON and TUB. Final BW and ADG tended (*P* ≤ 0.10) to be greater for FORCE compared to TUB and CON by 5.4% and 9.4%, respectively, G:F did not differ by treatment (*P* = 0.28). In the study conducted by Hamilton et al. (2021), steers were supplemented with the same stress tub used on this trial (Purina Animal Nutrition, St. Louis, MO, USA) during the first 21 day of a 42-day feedlot receiving phase and reported a tendency for 18.5% improvement on ADG for steers that received the stress tub supplementation. However, there were no significant differences observed. Berrett et al. (2015) reported no significant differences in feedlot performance between groups that received no added TM and those supplemented with Cu, Zn, Mn, I, Se, and Co at varying concentrations (10, 30, 20, 0.50, 0.10, 0.10 mg/kg of DM or 20, 100, 50, 0.50, 0.20, and 0.20 mg of mineral/kg of DM, respectively) for feedlot steers. Use of a YCP have been shown to enhance total tract digestibility of organic matter and increase mineral retention by the animal (Cole et al., 1992; Leicester et al., 2016). Whether enhanced growth performance for the stress-pack was due to the additional organic trace mineral supplementation or yeast culture product is not known. Relevant data of a receiving period meta-analysis suggest that while YCP did not influence DMI, it exhibited significant positive effects on both ADG and G:F by 5.8% and 2%, respectively, demonstrating its potential as a promising option to enhance cattle performance during the receiving period (Wagner et al., 2016). In contrast, Deters et al. (2018) observed no difference in performance when YCP was fed to receiving beef steers at a rate of 14 or 28 g·steer^−1^·d^−1^. Furthermore, the administration of YCP at 28.4 g·steer^−1^·d^−1^ had no effect on growth performance of shipping-stressed calves (Zinn et al.,1999).

**Table 4.** Cumulative effects of use and delivery method of supplemental organic trace minerals and a yeast culture product “stress pack” in newly weaned steer calves on growth performance from day 1 to 28, 29 to 49, and cumulative

	Treatments^1^				
Item	CON	FORCE	TUB	SEM	*P-*value
Pens, *n*	8	8	8	--	--
Steers, *n*	64	64	64	--	--
Initial BW^2,^ kg	257	256	257	0.5	0.17
Initial to d 28					
d 28 BW^3^, kg	283	286	285	3.3	0.19
ADG^4^, kg	0.94	1.06	0.99	0.114	0.10
DMI^5^, kg	5.34	5.57	5.36	0.132	0.01
As-fed tub intake, kg/d	*—*	*—*	0.12	*—*	*—*
ADG/DMI	0.18	0.19	0.19	0.0085	0.23
d 29^3^ to d 49					
ADG, kg	1.20	1.28	1.14	0.219	0.37
DMI, kg	6.79	7.10	6.64	0.279	0.01
ADG/DMI	0.177	0.180	0.171	0.0088	0.74
Initial to d 49					
Final BW^3^, kg	308^h^	313^g^	309^h^	2.1	0.10
ADG, kg	1.05^h^	1.16^g^	1.06^h^	0.100	0.07
DMI, kg	5.96^b^	6.22^a^	5.91^b^	0.158	0.01
ADG/DMI	0.176	0.185	0.179	0.0058	0.28

^1^No “stress pack” (CON), “stress-pack” directly fed in the diet (FORCE; Organic trace minerals (Availa 4, ZINPRO, Eden Prairie, MN) and a *Saccharomyces cerevisiae* yeast culture product (Diamond V XPC, Diamond V, Cedar Rapids), or cooked molasses stress tub (TUB; Stress Tub; Purina Animal Nutrition, St. Louis, MO, USA).

^2^No shrink was applied to initial BW.

^3^A 4% pencil shrink was applied to BW captured on d 28 and 49 to account for digestive tract fill.

^4^Average daily gain.

^5^Dry matter intake of total mixed ration (tub intake not included).

^a,b^Means within a row differ (*P* ≤ 0.05).

^g,h^Means within a row tend to differ (*P* ≤ 0.10).

**Figure 2.**
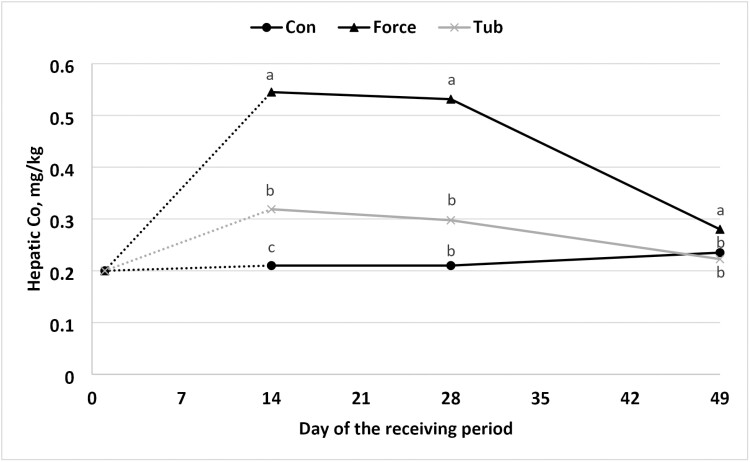
Hepatic concentration of cobalt in newly weaned steers initial to 49-d post weaning. No “stress pack” (CON), “stress-pack” directly fed in the diet (FORCE; Organic trace minerals (Availa 4, ZINPRO, Eden Prairie, MN) and a *Saccharomyces cerevisiae* yeast culture product (Diamond V XPC, Diamond V, Cedar Rapids), or cooked molasses stress TUB (TUB; Stress TUB; Purina Animal Nutrition, St. Louis, MO, USA). *P*-values: Treatment × day = 0.01; day = 0.01; treatment = 0.01; standard error of the mean = 0.030. a, b means within a day lacking a common superscript differ (*P *≤ 0.05). Abbreviations: CON = black circle; FORCE = black triangle; TUB = gray ×.

**Figure 3.**
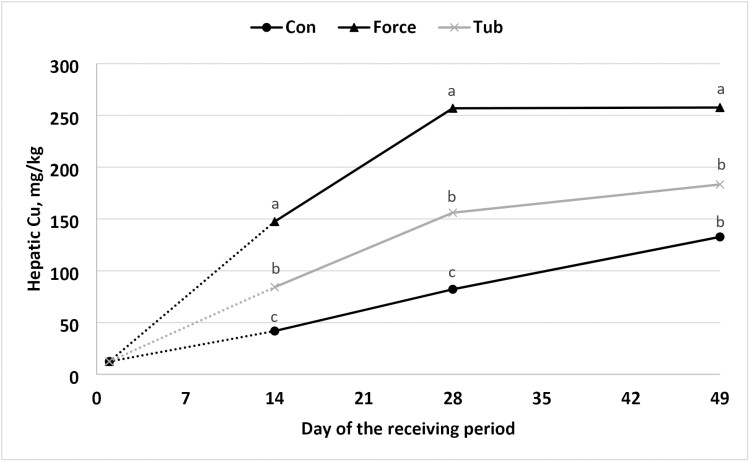
Hepatic concentration of copper in newly weaned steers initial to 49-d post weaning. No “stress pack” (CON), “stress-pack” directly fed in the diet (FORCE; Organic trace minerals (Availa 4, ZINPRO, Eden Prairie, MN) and a *Saccharomyces cerevisiae* yeast culture product (Diamond V XPC, Diamond V, Cedar Rapids), or cooked molasses stress TUB (TUB; Stress TUB; Purina Animal Nutrition, St. Louis, MO, USA). *P*-values: Treatment × day = 0.01; day = 0.01; treatment = 0.01; standard error of the mean = 19.1. a, b means within a day lacking a common superscript differ (*P *≤ 0.05). Abbreviations: CON = black circle; FORCE = black triangle; TUB = gray ×.

In the present study, a treatment × day interaction (*P* ≤ 0.01), for hepatic concentrations of Co and Cu was noted (Figures 2 and 3). The steers on FORCE treatment had greater hepatic Co (*P* ≤ 0.05) compared to TUB and CON on day 14, 28, and 49. However, hepatic Co concentration was greater for TUB compared to CON on day 14 but did not differ on day 28 and day 49. Hamilton et al. (2021) and Ranches et al. (2021) also found improvements of hepatic TM concentrations when offered molasses blocks fortified with TM for beef calves. Ranches et al. (2021) found enhancements in hepatic TM concentration (Cu, Co, and Mn; *P* ≤ 0.01) when supplemented early weaned calves with a molasses block at the concentration of 0.15, 550, 1,650, and 2,200 mg/kg of Co, Cu, Zn, and Mn, respectively. Hamilton et al. (2021) noted that supplemented steers had greater hepatic Co during the treatment period (day 1 to day 21); however, from day 21 to day 42, Co hepatic concentration did not differ from CON. Similar effects were observed in the current experiment where steers from TUB had greater hepatic Co compared to CON on day 14 (*P* < 0.05), but hepatic Co content was similar to CON on days 28 and 49 (*P* > 0.10).

Steers from FORCE treatment had greater hepatic Cu (*P* ≤ 0.05) compared to TUB and CON on days 14, 28, and 49. This is likely driven by greater DMI consumed by the FORCE treatment compared to the other treatments thus leading to greater overall daily Cu intake. As observed when an organic source of TM was fed, Rhoads et al. (2003) and Marques et al. (2016), demonstrated increased hepatic Cu stores with increased dietary concentrations of Cu. Steers from TUB had greater hepatic Cu compared to those in the CON group on days 14 and 28 (*P* < 0.05), but hepatic Cu content was similar to CON on day 49 (*P *> 0.10). This trend is consistent with the findings of Hamilton et al. (2021), where during the initial 21 days of supplementation, cattle in the supplemented group had higher hepatic Cu levels. However, on day 42, hepatic Cu concentrations in the CON group did not differ to those in the supplemented group.

**Figure 4.**
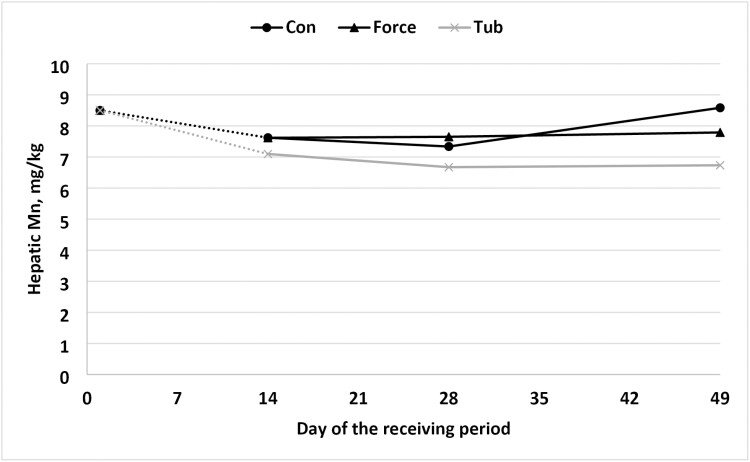
Hepatic concentration of manganese in newly weaned steers initial to 49-d post weaning. No “stress pack” (CON), “stress-pack” directly fed in the diet (FORCE; Organic trace minerals (Availa 4, ZINPRO, Eden Prairie, MN) and a *Saccharomyces cerevisiae* yeast culture product (Diamond V XPC, Diamond V, Cedar Rapids), or cooked molasses stress TUB (TUB; Stress TUB; Purina Animal Nutrition, St. Louis, MO, USA). *P*-values: Treatment × day = 0.24; day = 0.04; treatment = 0.07; standard error of the mean = 0.41. a, b means within a day lacking a common superscript differ (*P* ≤ 0.05). Abbreviations: CON = black circle; FORCE = black triangle; TUB = gray ×.

**Figure 5.**
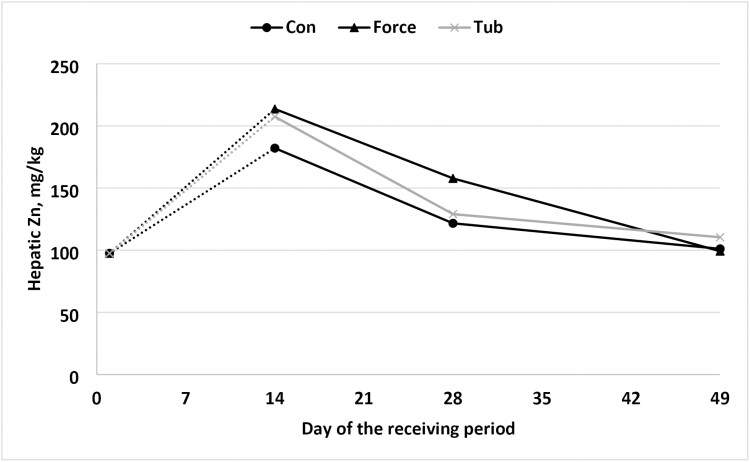
Hepatic concentration of zinc in newly weaned steers initial to 49-d post weaning. No “stress pack” (CON), “stress-pack” directly fed in the diet (FORCE; Organic trace minerals (Availa 4, ZINPRO, Eden Prairie, MN) and a *Saccharomyces cerevisiae* yeast culture product (Diamond V XPC, Diamond V, Cedar Rapids), or cooked molasses stress TUB (TUB; Stress TUB; Purina Animal Nutrition, St. Louis, MO, USA). *P*-values: Treatment × day = 0.09; day = 0.01; treatment = 0.50; standard error of the mean = 15.2. a, b means within a day lacking a common superscript differ (*P *≤ 0.05). Abbreviaitons: CON = black circle; FORCE = black triangle; TUB = gray ×.

In the present study, a tendency for a treatment × day interaction was observed for hepatic concentration of Mn (*P* = 0.07) and Zn (*P* = 0.09). In addition, a day effect was noted (*P* = 0.04; *P* = 0.01) for Mn and Zn hepatic concentration, respectively (Figures 4 and 5). Differing from Hamilton et al. (2021) where a treatment × day interaction was found on day 7 for Zn hepatic concentration, being 27% higher when compared to CON steers. However, on day 21 and day 42 hepatic concentrations of Zn did not differ. Furthermore, the hepatic Cu concentration of the steers of the present study was considerably higher when compared with Hamilton et al. (2021). This disparity may have implications for Zn availability, as Cu is known to be a strong antagonist to Zn (Radwinska and Zarczynska, 2014). However, hepatic Zn did not differ between treatment groups.

## Conclusion

Providing TM supplementation for calves through limit-fed creep feeding, injectable trace minerals, or low moisture cooked molasses blocks improves trace mineral status of cattle (Arthington et al., 2014; Caramalac et al., 2017; Hamilton et al., 2021; Ranches et al., 2021).

Using the cooked-molasses based block to provide additional TM and YCP has the benefit of labor savings compared to other supplementation methods (Kunkle et al., 2000). However, a major issue with free choice supplementation is over or under consumption of the product (Hamilton et al., 2021), in the present experiment steers offered the cooked-tub under consumed the supplement, which can be a factor that explains the poorer performance compared to FORCE. The FORCE treatment resulted in the best performance and greater Co and Cu hepatic concentrations compared to CON and TUB. Steers from TUB had greater hepatic concentrations of Co and Cu on day 14 (Co), and 14 and 28 (Cu), respectively. In summary, the application of a “stress-pack” in diets offered to newly weaned cattle: 1) enhanced hepatic concentrations of Co and Cu and 2) increased production responses, but method of delivery influences DMI and tended to influence daily gain.

## Data Availability

Data can be made available with reasonable request to ZKS.
